# Supplementary material for: Acquired resistance to PD-L1 inhibition enhances a type I IFN-regulated secretory program in tumors
Source: EMBO Rep. 2024 Dec 11;26(2):521–59. doi: 10.1038/s44319-024-00333-0 (PMC11772817; doi:10.1038/s44319-024-00333-0)
Supplement: Supplementary file 1 — Appendix [file 44319_2024_333_MOESM1_ESM.pdf]

APPENDIX FILES FOR:

‘Acquired resistance to PD-L1 inhibition enhances a type I IFN-regulated secretory program in tumors’

Shi *et al.*

TABLE OF CONTENTS

A. APPENDIX FIGURES 3

Appendix Figure S1: Cytokine Antibody Array Replicates and Exposure from Figure 2C 3

Appendix Figure S2: Cytokine Antibody Array layout 4

Appendix Figure S3: Western blotting replicates as uncropped unaltered images (related to Figure 2E). 5

Appendix Figure S4: IFNβ protein expression in EMT6-P/PDR cells 6

Appendix Figure S5: Type II IFNγ stimulation regulate PTIS after acquired PD-L1 resistance 7

Appendix Figure S6: Western blotting replicates as uncropped unaltered images (related to Figure 4F) 9

Appendix Figure S7: Anti-IL6 treatment in EMT6-P/PTR and IFNAR1KD 10

Appendix Figure S8: Western blotting replicates as uncropped unaltered images (related to Appendix Figure S7) 11

Appendix Figure S9: Cytokine Antibody Array Replicates and Exposure from Figure 5A 12

Appendix Figure S10: GSEA of JAK/STAT pathways in EMT6-P/PTR tumors 13

B. APPENDIX TABLES 14

Appendix Table S1: C2 Curated Pathways Identified via GSEA Analysis 14

Appendix Table S2: Published and Hallmark gene sets used for Gene Set Enrichment Analysis (GSEA) 15

Appendix Table S3: List of PTIS and $PTIS^{DOWN}$ Genes 16

Appendix Table S4: JAK/STAT Pathways Identified via GSEA Analysis 18

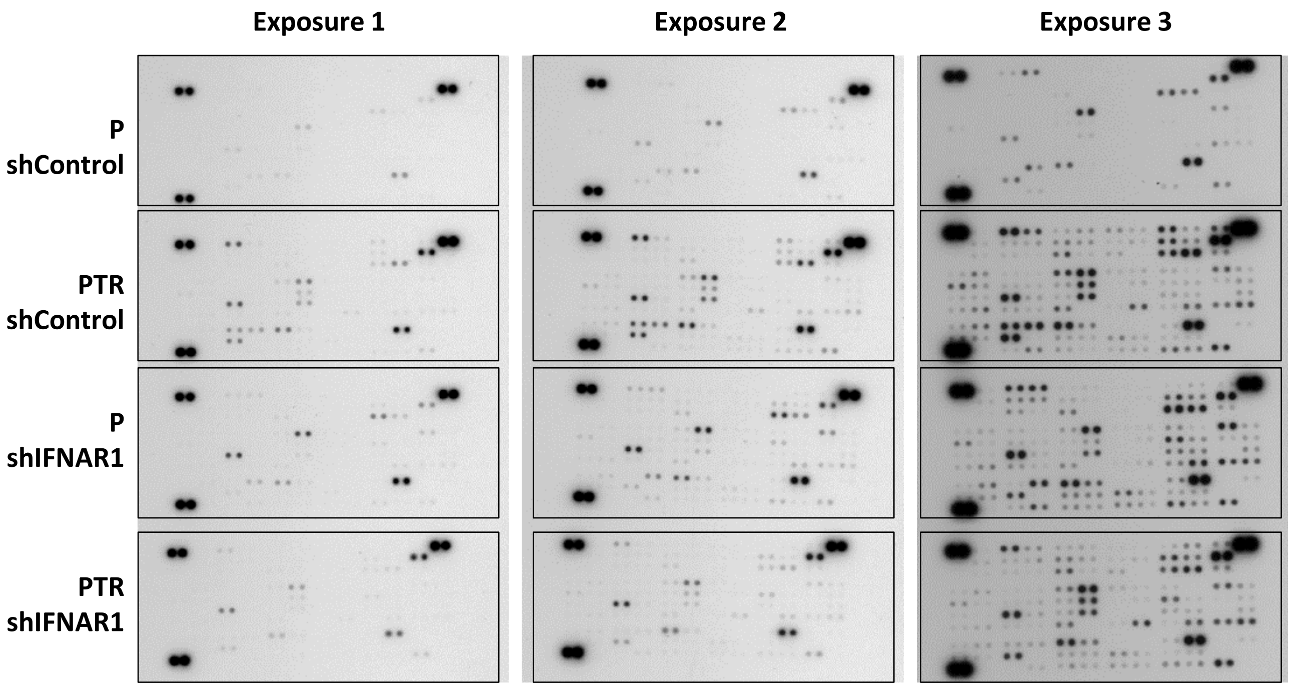

<u>Appendix Figure S1</u>: Cytokine Antibody Array Replicates and Exposure from Figure 2C

Overlay Legend

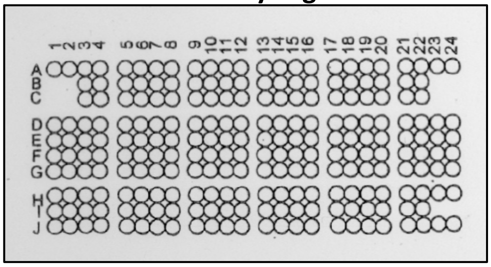

Cytokine Antibody Array Legend

A1	Positive	C5		E5	Flt-3	G3		I1	
A2	Control	C6	Chemerin	E6	Ligand	G4	IL-11	I2	PDGF-BB
A3	Adiponecti	C7	Chitinase 3-	E7		G5		I3	Pentraxin
A4	n/ Acrp30	C8	like 1	E8	Gas 6	G6	IL-12 p40	I4	2/ SAP
A5	Amphiregu	C9	Coagulation	E9		G7		I5	Pentraxin
A6	lin	C10	Factor III/	E10	G-CSF	G8	IL-13	I6	3/ TSG-14
A7	Angiopoiet	C11	Complemen	E11		G9		I7	Periostin/
A8	in-1	C12	t	E12	GDF-15	G10	IL-15	I8	OSF-2
A9	Angiopoiet	C13	Complemen	E13		G11		I9	Pref-1/ DLK-
A10	in-2	C14	t Factor D	E14	GM-CSF	G12	IL-17A	I10	1/ FA1
A11	Angiopoiet	C15	C-Reactive	E15		G13		I11	
A12	in-like	C16	Protein/	E16	HGF	G14	IL-22	I12	Proliferin
A13	BAFF/	C17	CX3CL1/	E17	ICAM-1/	G15		I13	Proprotein
A14	BLyS/	C18	Fractalkine	E18	CD54	G16	IL-23	I14	Convertase
A15		C19		E19		G17		I15	
A16	C1q	C20	CXCL1/ KC	E20	IFN-y	G18	IL-27 p28	I16	RAGE
A17	CCL2/ JE/	C21	CXCL2/ MIP-	E21		G19		I17	
A18	MCP-1	C22	2	E22	IGFBP-1	G20	IL-28A/ B	I18	RBP4
A19	CCL3/	D1		E23		G21		I19	
A20	CCL4/ MIP-	D2	CXCL9/ MIG	E24	IGFBP-2	G22	IL-33	I20	Reg3G
A21	CCL5/	D3	CXCL10/ IP-	F1		G23		I21	
A22	RANTES	D4	10	F2	IGFBP-3	G24	LDL R	I22	Resistin
A23	Positive	D5	CXCL11/ I-	F3		H1		J1	Positive
A24	Control	D6	TAC	F4	IGFBP-5	H2	Leptin	J2	Control
B3		D7	CXCL13/	F5		H3		J3	E-Selectin/
B4	CCL6/ C10	D8	BLC/ BCA-1	F6	IGFBP-6	H4	LIF	J4	CD62E
B5	CCL11/	D9		F7	IL-1a/ IL-	H5	Lipocalin-	J5	P-Selectin/
B6	Eotaxin	D10	CXCL16	F8	1F1	H6	2/ NGAL	J6	CD62P
B7	CCL12/	D11		F9	IL-1ß/ IL-	H7		J7	Serpin E1/
B8	MCP-5	D12	Cystatin C	F10	1F2	H8	LIX	J8	PAI-1
B9	CCL17/	D13		F11	IL-1ra/ IL-	H9		J9	Serpin F1/
B10	TARC	D14	DKK-1	F12	1F3	H10	M-CSF	J10	PEDF
B11	CCL19/	D15	DPPIV/	F13		H11		J11	Thrombop
B12	MIP-3ß	D16	CD26	F14	IL-2	H12	MMP-2	J12	oietin
B13	CCL20/	D17		F15		H13		J13	TIM-1/ KIM-
B14	MIP-3a	D18	EGF	F16	IL-3	H14	MMP-3	J14	1/ HAVCR
B15	CCL21/	D19	Endoglin/	F17		H15		J15	
B16	6Ckine	D20	CD105	F18	IL-4	H16	MMP-9	J16	TNF-a
B17	CCL22/	D21		F19		H17	Myelopero	J17	VCAM-1/
B18	MDC	D22	Endostatin	F20	IL-5	H18	xidase	J18	CD106
B19		D23		F21		H19	Osteoponti	J19	
B20	CD14	D24	Fetuin	F22	IL-6	H20	n (OPN)	J20	VEGF
B21	CD40/	E1		F23		H21	Osteoprot	J21	WISP-1/
B22	TNFRSF5	E2	FGF acidic	F24	IL-7	H22	egerin/	J22	CCN4
C3		E3		G1		H23	PD-ECGF/	J23	Negative
C4	CD160	E4	FGF-21	G2	IL-10	H24	Thymidine	J24	Control

Appendix Figure S2: Cytokine Antibody Array layout

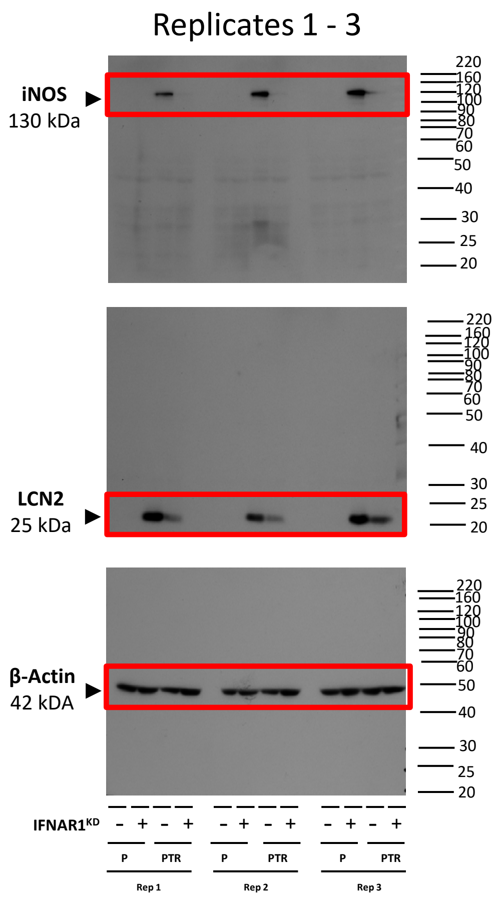

<u>Appendix Figure S3</u>: Western blotting replicates as uncropped unaltered images (related to Figure 2E).

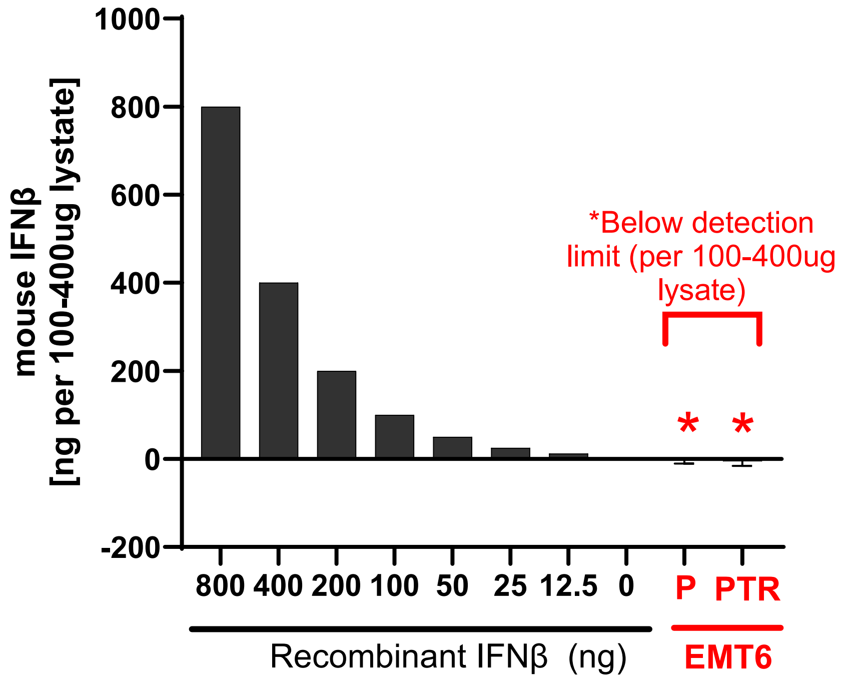

Appendix Figure S4: IFNβ protein expression in EMT6-P/PDR cells. N=1

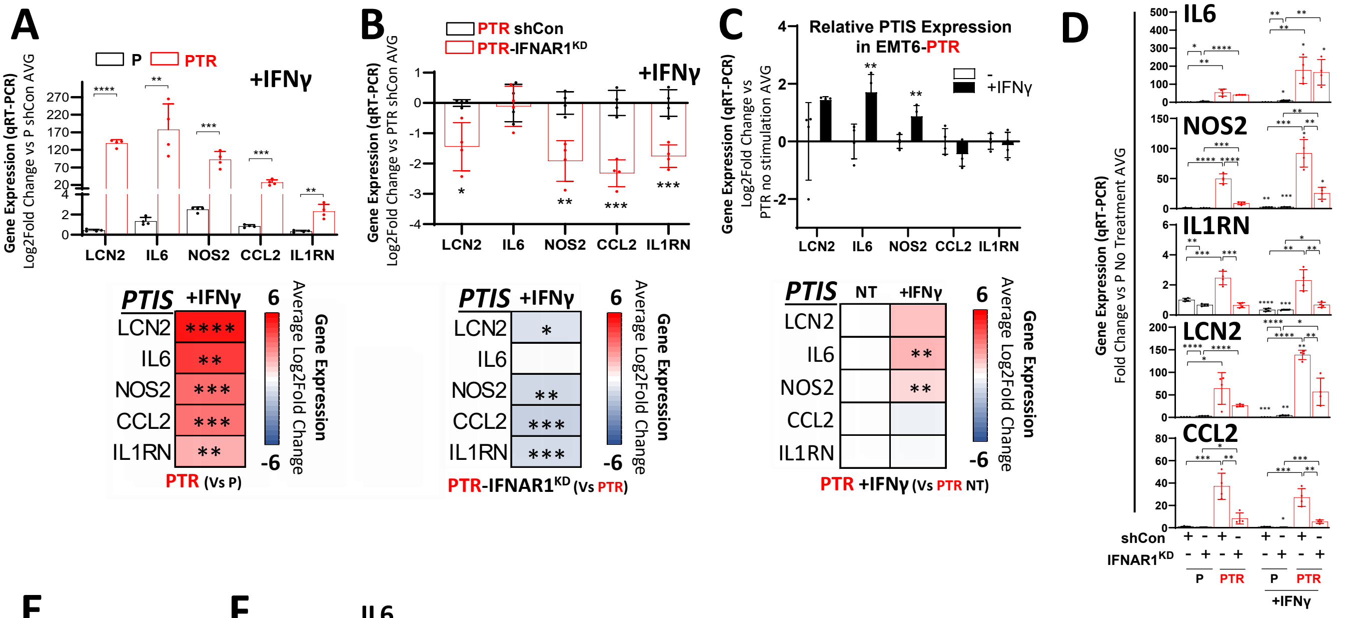

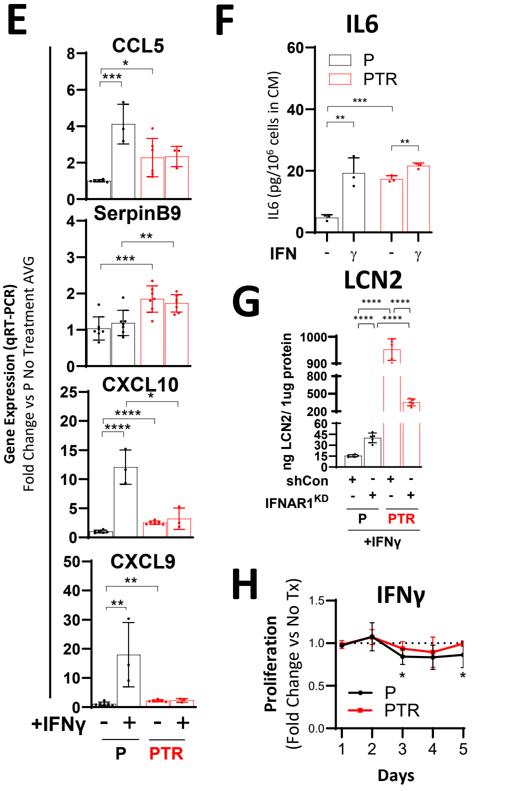

I

PTIS		-			+IFNγ			
	P		PTR		P		PTR	
	shCon	IFNAR1KD	shCon	IFNAR1KD	shCon	IFNAR1KD	shCon	IFNAR1KD
IL6	vs P	*	**	****		***	**	**
NOS2			****	***	**	**	***	**
IL1RN		**	***	*	****	****	*	*
LCN2		****	*	****	***	****	****	*
CCL2			***	*			***	**
IL6	vs P + IFNγ					**	**	**
NOS2							***	**
IL1RN							**	*
LCN2						****	****	**
CCL2							***	**
IL6	vs P	*	vs PTR		vs P + IFNγ	**	vs PTR + IFNγ	
NOS2				****				**
IL1RN		**		***				**
LCN2		****				****		**
CCL2				**				**

Increase
Decrease

<u>Appendix Figure S5:</u> Type II IFNγ stimulation modulates PTIS and ISG expression after acquired PD-L1 resistance

(A) PTIS factor expression increased in EMT6-PTR cells after 48 hr stimulation with IFNγ relative to EMT6-P controls. Represented as bar graph (top) and heatmap (bottom). qRT-PCR, n=4, statistics performed via two tailed t-test.

(B) IFNγ-enhanced PTIS factor expression reversed in EMT6-PTR-IFNAR1^{KD} cells. Shown as relative to PTR controls after 48 hr IFNγ stimulation and represented as bar graph (top) and heatmap (bottom). qRT-PCR, n=4, statistics performed via two tailed t-test.

(C) PTIS factor expression in EMT6-PTR cells after IFNγ stimulation enhanced for IL6 and NOS2. Data shown as relative to unstimulated EMT6-PTR cells and represented as bar graph (top) and heatmap (bottom). qRT-PCR, n=4, statistics performed via two tailed t-test.

(D) PTIS factor expression in of EMT6-P and -PTR cells before and after knockdown of IFNAR1^{KD}, and after IFNγ stimulation shown as bar plots. These are the full datasets for Appendix Figures S5A, S5B, S5C. qRT-PCR, n=4, statistics performed via two tailed t-test.

(E) Assessment of other ISGs showed some consistency with increased expression in EMT6-PTR compared to EMT6-P controls for SerpinB9 but other ISGs were unchanged or had decreased expression after IFNγ stimulation. Data shown as bar plots. qRT-PCR, n=3-7, statistics performed via two tailed t-test.

(F-G) IFNγ stimulated gene elevations in EMT6-PTR cells were confirmed in conditioned media and cell lysates using protein ELISAs for IL6 (Appendix Figure S5F) and LCN2 (Appendix Figure S5G), respectively. ELISA, n=3, statistics performed via two tailed t-test.

(G) IFNγ stimulated LCN2 protein increase EMT6-PTR cell lysates were reversed following knockdown of IFNAR1^{KD}. Data shown as bar plots. ELISA, n=5, statistics performed via two tailed t-test.

(H) Control experiments showed EMT6-P and EMT6-PTR cells did not consistently respond differently to IFNγ anti-proliferative effects. Cells treated with IFNγ for 5 days and proliferation measured daily by MTS. n=10, statistics performed via two tailed t-test.

(I) Table summarizing two tailed t-test statistical comparisons for qRT-PCR data shown in Appendix Figures 4 and S3.

Data Information: Parental (P); αPD-L1 Treatment-Resistant (PTR); IFN stimulated genes (ISGs); IFNAR1 knockdown (IFNAR1^{KD}); shRNA vector control (shCon; shown here as a '-'); Cells were treated with 10ng/ml of IFNγ and collected after 5 days. for proliferation experiments cells were treated with 10ng/ml of IFNs starting on day 0 and treatment and fresh media was replaced on day 3. $p<0.05$, ** $p<0.01$, *** $p<0.001$, **** $p<0.0001$ indicate significance compared untreated controls unless otherwise shown (lines). See Appendix Source Data for exact p values. Bar graphs and line graphs show mean ± SD. All replicates shown represent technical replicates unless otherwise specified.*

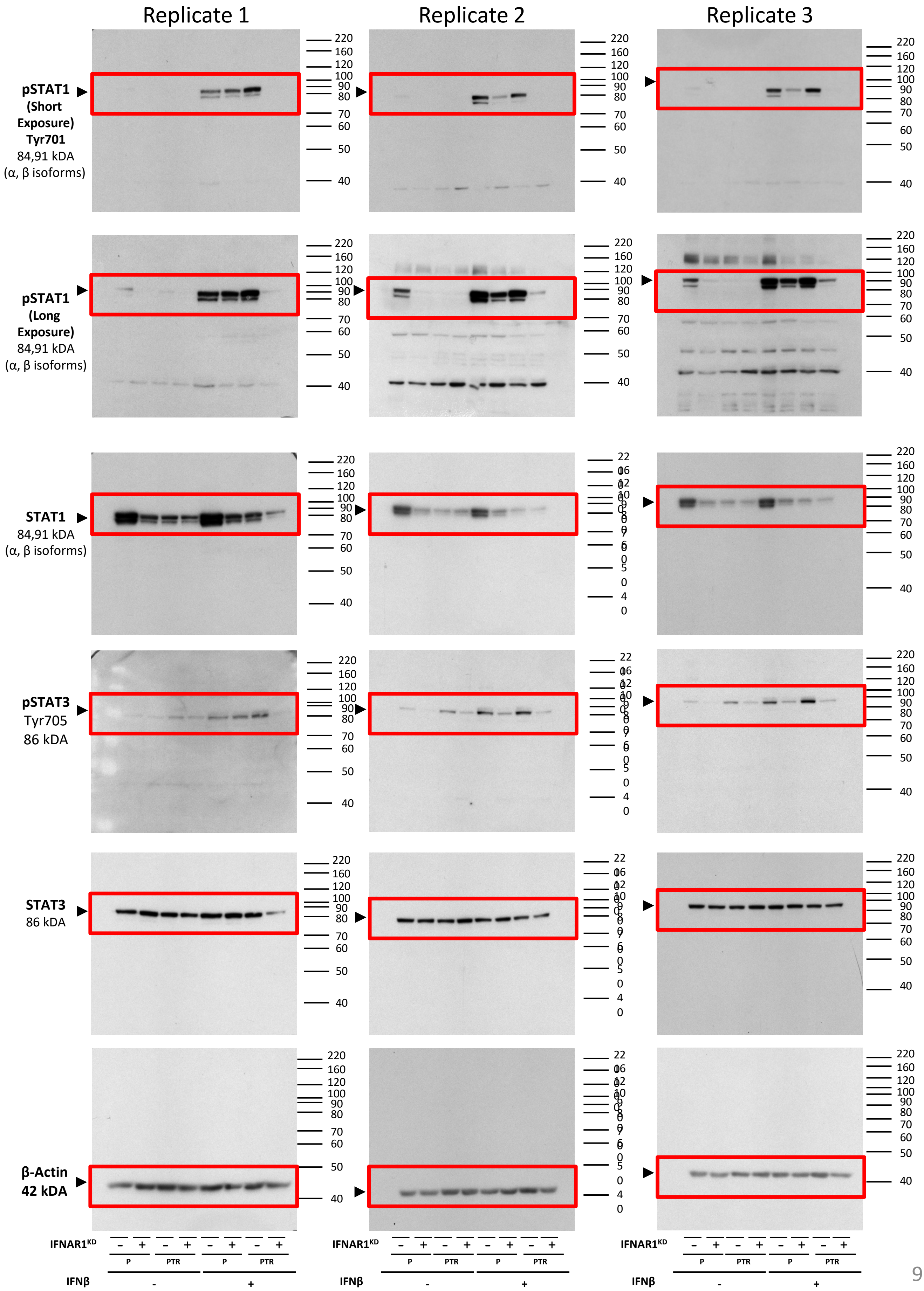

Appendix Figure S6 : Western blotting replicates as uncropped unaltered images (related to Figure 4F)

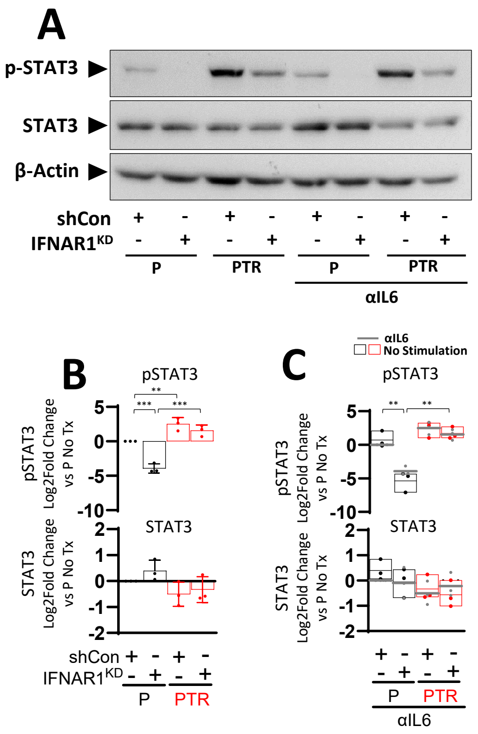

<u>Appendix Figure S7</u>: αIL6 treatment in EMT6-P/PTR and IFNAR1KD

(A) Phosphorylated and total levels of STAT3 in lysates of EMT6-P and -PTR before and after knockdown of IFNAR1 following αIL6 treatment (Western Blot).

(B-C) Densitometry quantification of western blots shown in **(A)** representing relative phosphorylated STAT3 compared to total STAT3 at **(B)** baseline and **(C)** after αIL6 treatment. n=3, statistics performed via two tailed t-test.

*Data Information: Parental (P); Treatment (Tx); αPD-L1 Treatment-Resistant (PTR); Conditioned Media (CM); IFN stimulated genes (ISGs); IFNAR1 knockdown (IFNAR1^{KD}); shRNA vector control (shCon); Cells were treated with 10μg/ml of αIL6 for 48 hours and collected for STAT1/3 westerns. * p<0.05, ** p<0.01, *** p<0.001, **** p<0.0001 indicate significance compared untreated controls unless otherwise shown (lines). See Appendix Source Data for exact p values. Bar graphs show mean ± SD. Box plots indicate range between minimum and maximum, central line depicts the mean. All replicates shown represent technical replicates unless otherwise specified.*

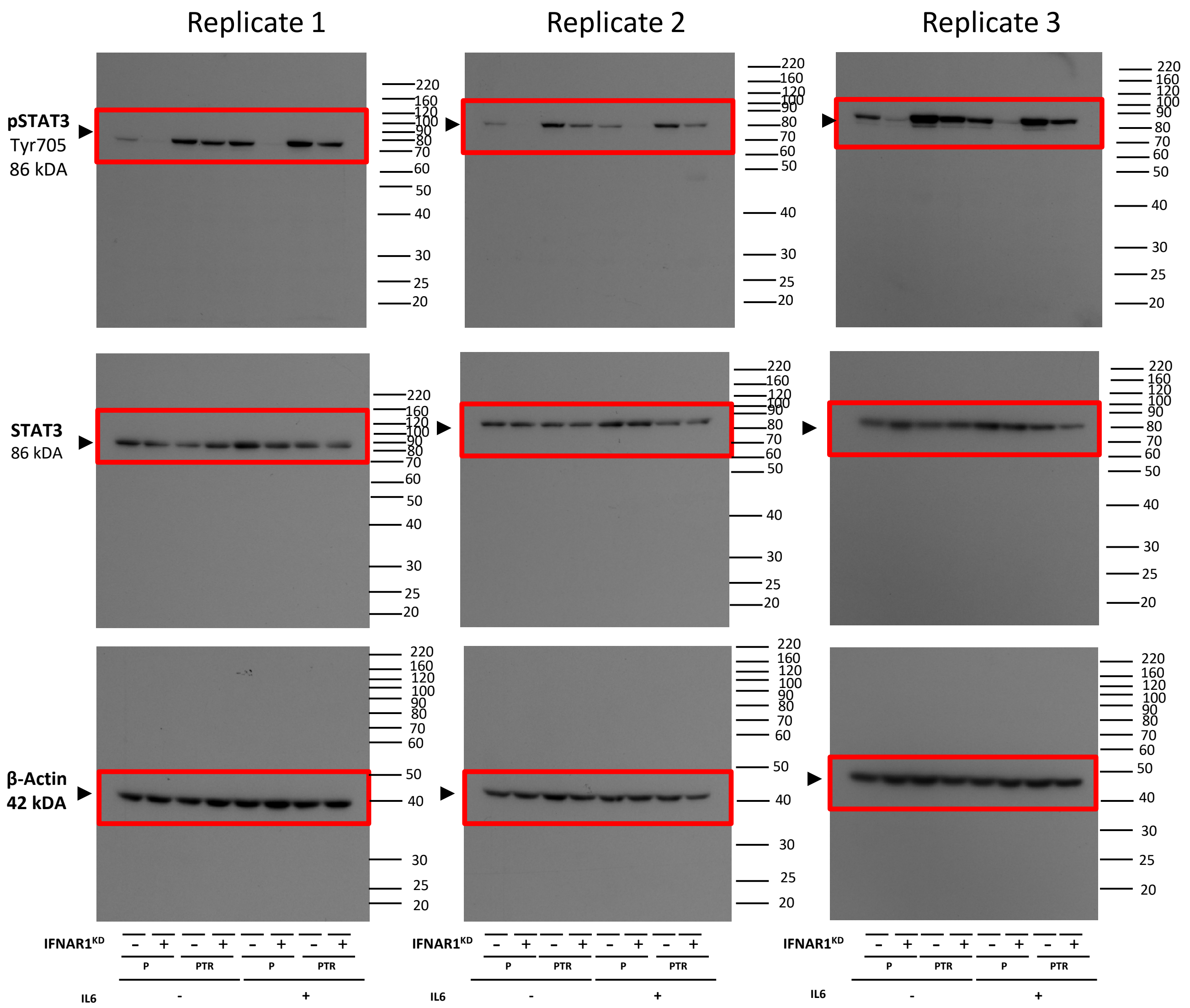

<u>Appendix Figure S8 :</u> Western blotting replicates as uncropped unaltered images (related to Appendix Figure S7)

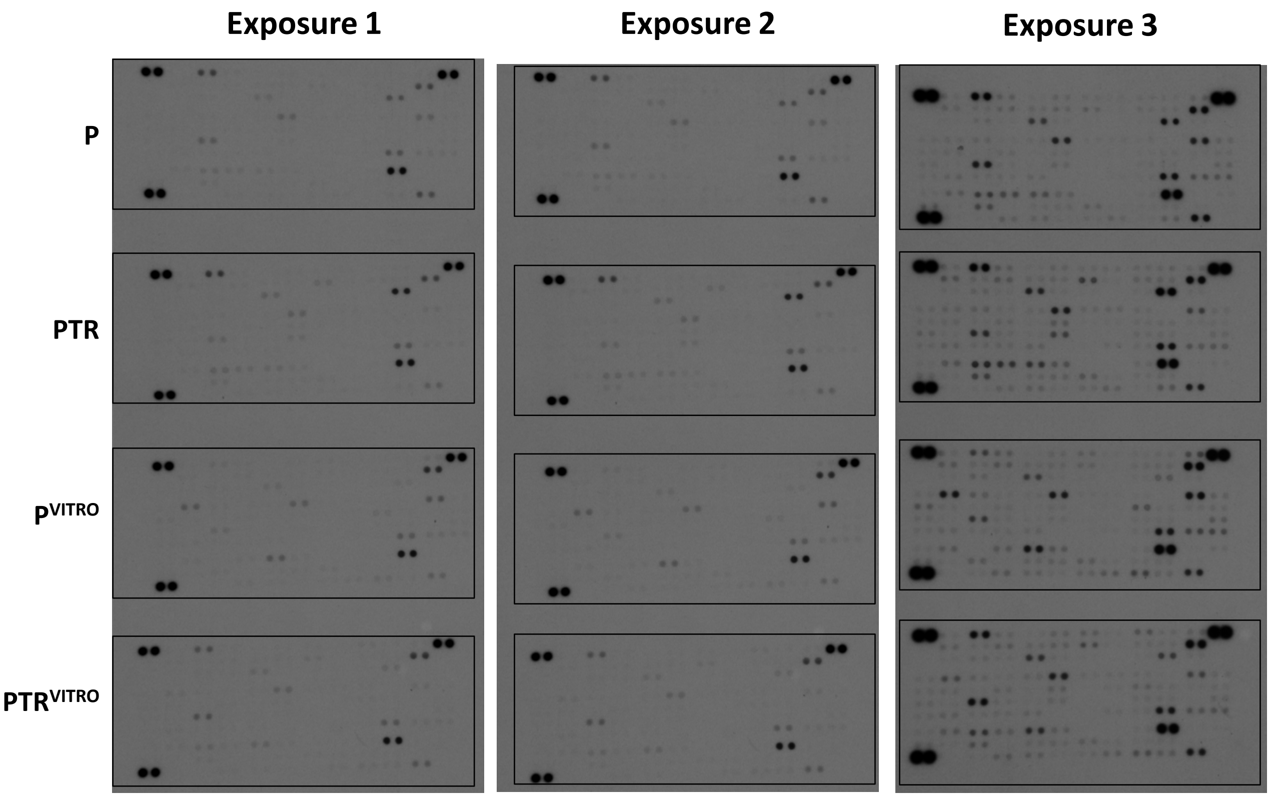

Appendix Figure S9: Cytokine Antibody Array Replicates and Exposure from Figure 5A

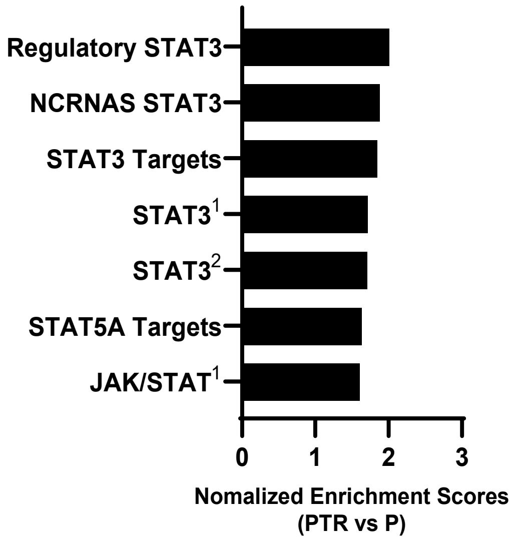

Appendix Figure S10: **GSEA of JAK/STAT pathways in EMT6-P/PTR tumors**

B. APPENDIX TABLES

Appendix Table S1: C2 Curated Pathways Identified via GSEA Analysis

Annotated Name	Full Name	Systematic Name
Stroma	DURAND_STROMA_S_UP	M2581
ECM GP	NABA_ECM_GLYCOPROTEINS	M3008
ECM Receptor	KEGG_ECM_RECEPTOR_INTERACTION	M7098
Cancer Microenv	NAKAMURA_CANCER_MICROENVIRONMENT_UP	M7581
ECM PG	REACTOME_ECM_PROTEOGLYCANS	M27219
miRNA in ECM	WP_MIRNA_TARGETS_IN_ECM_AND_MEMBRANE_RECEPTORS	M39565
ECM Affiliated	NABA_ECM_AFFILIATED	M5880
miR5093P ECM	WP_MIR5093P_ALTERATION_OF_YAP1ECM_AXIS	M39421
EGFR 24hr	KOBAYASHI_EGFR_SIGNALING_24HR_UP	M10290
IGF1/AKT	WP_FACTORS_AND_PATHWAYS_AFFECTING_INSULINLIKE_GROWTH_FACTOR_IGF1AKT_SIGNALING	M39569
FGFR2	REACTOME_FGFR2_LIGAND_BINDING_AND_ACTIVATION	M27062
FGFR2B	REACTOME_FGFR2B_LIGAND_BINDING_AND_ACTIVATION	M27066
FGFR1mut	REACTOME_FGFR1_MUTANT_RECEPTOR_ACTIVATION	M661
EGFR 6Hr	KOBAYASHI_EGFR_SIGNALING_6HR_UP	M7331
FGFR1 Fusion mut	REACTOME_SIGNALING_BY_CYTOSOLIC_FGFR1_FUSION_MUTANTS	M673
TGFB1	PLASARI_TGFB1_SIGNALING_VIA_NFIC_10HR_UP	M2452
VEGFA	WESTON_VEGFA_TARGETS_6HR	M1521
FGFR1 in disease	REACTOME_SIGNALING_BY_FGFR1_IN_DISEASE	M27536
IGF1R	REACTOME_SIGNALING_BY_TYPE_1_INSULIN_LIKE_GROWTH_FACTOR_1_RECEPTOR_IGF1R_	M27168
IFNA[1]	MOSERLE_IFNA_RESPONSE	M3218
IFNγ[1]	SANA_RESPONSE_TO_IFNG_UP	M4551
STAT3[1]	WP_REGULATORY_CIRCUITS_OF_THE_STAT3_SIGNALING_PATHWAY	M39824
IFN Anti-Viral	BOSCO_INTERFERON_INDUCED_ANTIVIRAL_MODULE	M2532
STAT3[2]	ST_STAT3_PATHWAY	M9174
IFNγ[2]	REACTOME_INTERFERON_GAMMA_SIGNALING	M965
STAT3[3]	BIOCARTA_STAT3_PATHWAY	M22063
IFNA[2]	BIOCARTA_IFNA_PATHWAY	M22056
STAT3[4]	WIERENGA_STAT5A_TARGETS_GROUP1	M2215
JAK/STAT	ST_JAK_STAT_PATHWAY	M5248
TNSF11	FUKUSHIMA_TNFSF11_TARGETS	M2207
IFNγ[3]	ST_INTERFERON_GAMMA_PATHWAY	M4170

Appendix Table S2: Published and Hallmark gene sets used for Gene Set Enrichment Analysis (GSEA)

Gene Sets		Number of Genes	Systematic Name or PMID	Link
Published	Liu et al 2018	38	30559422	https://pubmed.ncbi.nlm.nih.gov/30559422/
	Benci et al. 2019 ISG.RS	38	31398344	https://pubmed.ncbi.nlm.nih.gov/31398344/
	Benci et al. 2019 IFNG.GS	176	31398344	https://pubmed.ncbi.nlm.nih.gov/31398344/
	Weichselbaum et al. 2008	49	19001271	https://pubmed.ncbi.nlm.nih.gov/19001271/
	Thorsson et al. 2018	24	29628290	https://pubmed.ncbi.nlm.nih.gov/29628290/
	Higgs et al. 2018	21	29716923	https://pubmed.ncbi.nlm.nih.gov/29716923/
GSEA MSigDB	Hallmark IFNA	97	M5911	https://www.gsea-msigdb.org/gsea/msigdb/cards/HALLMARK_INTERFERON_ALPHA_RESPONSE
	Hallmark IFNγ	200	M5913	https://www.gsea-msigdb.org/gsea/msigdb/cards/HALLMARK_INTERFERON_GAMMA_RESPONSE

Glossary: Molecular Signatures Database (MSigDB), Interferon stimulated genes resistance signature (ISG.RS), Interferon gamma hallmark gene set (IFNG.GS), Interferon alpha (IFNA), Interferon gamma (IFNγ)

Appendix Table S3: List of PTIS and $PTIS^{DOWN}$ Genes

Anti-PD-L1 Treatment Induced Secretome (PTIS)						
**PTIS (preliminary)**			**PTIS (enriched)**	**$PTIS^{DOWN}$ (preliminary)**		**$PTIS^{DOWN}$ (enriched)**
NOS2	KAZALD1	PRSS35	IL6	CKM	LDLR	ICAM1
LCN2	SERPINA3N	LUM	CCL2	MCPT8	CFP	CSF1
ANGPT1	GGT1	CDNF	CXCL10	MMP10	S100A3	COL18A1
COL10A1	CFH	ISLR	NOS2	COL4A5	SRPX2	HAVCR1
FAM180A	ADAMTS15	WNT6	CCL5	LGR6	ISM1	
WFDC1	SNED1	LBP	CXCL9	AREG	GPX7	
LGALS7	GPC6	SEMA6A	IFNB1	ADAMTS16	TNC	
ITIH2	LRRC17	SEMA6C	LCN2	MUC16	PRSS46	
MGP	ELN	C2	IGFBP6	CFD	CDCA8	
COMP	ART5	TNFAIP6	IL1RN	PF4	GFRA1	
CLEC3B	OGN	TRIL	CCL20	WNT5A	ULK4	
INHBE	COLQ	ADAMTS1	SERPINB9	OBSCN	SERPINE2	
COL11A1	CILP	HTRA3		TFPI2	MMP12	
IL33	KITL	VWF		CES2E	CXCL14	
APOD	GHR	MMP2		COL5A3	CCL12	
SPON2	PI15	ADNP		FGF21	GDF15	
FMOD	GBP10	ADAMTSL5		S100A7A	C1QTNF2	
ITGBL1	C8G	FGFBP1		CES1G	LRRN2	
EFEMP1	GDF3	PGLYRP1		ERFE	TGFB2	
THSD7A	LEPR	F3		TMPRSS11B	CPXM2	
CXCL13	LAMC2	EDN2		CCL22	S100A11	
OMD	COL3A1	LGALS4		COL17A1	FAM83D	
PRTN3	HBB-BS	ARSG		EREG	PODNL1	
2610528A11RIK	HPN	C1RL		SFN	OTOS	
AGER	SELP	DPYSL3		SERPINB2	PRC1	
S100B	HBA-A1	TNFSF10		CCL11	COL15A1	
AMY1	HHIP	POSTN		IL1RL1	WNT11	
CRISPLD1	MATN4	NXPE4		GREM1	FGF1	
DCN	SERPINA1B	LAMB2		PENK	ICAM1	
SEMA3E	HBA-A2	SAA3		HBEGF	CSF1	
ALPL	FAP	ECM2		LCAT	HAVCR1	
SECTM1B	GSTM7	C1S2		CEP55	COL18A1	
LGI4	TNXB	CD163		CCL7		
MMRN2	FBLN7	CCL5		CSF2		
ARSI	HBB-BT	CXCL9		MFAP5		
PRELP	HSPB6	IFNB1		MFAP2		
PKNOX2	SEMA3G	CXCL10		FREM1		
R3HDML	MATN3	IGFBP6		GREM2		
F5	SLC2A4	IL1RN		FABP5		
XPNPEP2	OLFML3	AREG		MMP9		
SERPINB1A	LOX	CCL20		MMP23		
EGF	SERPING1	VEGFA		PCOLCE2		

IGF1	WNT5B	CXCL16		MASP2		
ASPN	C4B	CCL2		TMPRSS11F		
		IL6				
		SERPINB9				

Appendix Table S4: JAK/STAT Pathways Identified via GSEA Analysis

Annotated Name	Full Name	Systematic Name
Regulatory STAT3	WP_REGULATORY_CIRCUITS_OF_THE_STAT3_SIGNALING	M39824
NCRNAS STAT3	WP_NCRNAS_INVOLVED_IN_STAT3_SIGNALING_IN_HCC	M39512
STAT3 Targets	DAUER_STAT3_TARGETS_DN	M13696
STAT3[1]	ST_STAT3_PATHWAY	M9174
STAT3[2]	BIOCARTA_STAT3_PATHWAY	M22063
STAT5A Targets	WIERENGA_STAT5A_TARGETS_GROUP1	M2215
JAK/STAT[1]	ST_JAK_STAT_PATHWAY	M5248
