# Supplementary figures and images for: Acquired resistance to PD-L1 inhibition enhances a type I IFN-regulated secretory program in tumors
Source: EMBO Rep. 2024 Dec 11;26(2):521–59. doi: 10.1038/s44319-024-00333-0 (PMC11772817; doi:10.1038/s44319-024-00333-0)

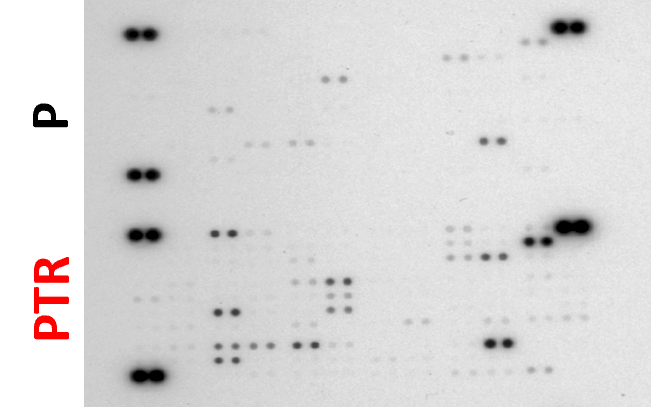

Supplement: Supplementary file 4 — Source data Fig. 2 [file 44319_2024_333_MOESM4_ESM.zip › Figure 2/2C/2C-left no annotations.png]

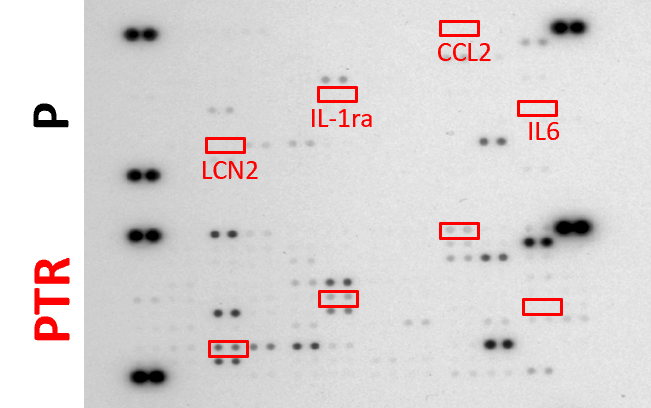

Supplement: Supplementary file 4 — Source data Fig. 2 [file 44319_2024_333_MOESM4_ESM.zip › Figure 2/2C/2C-left.png]

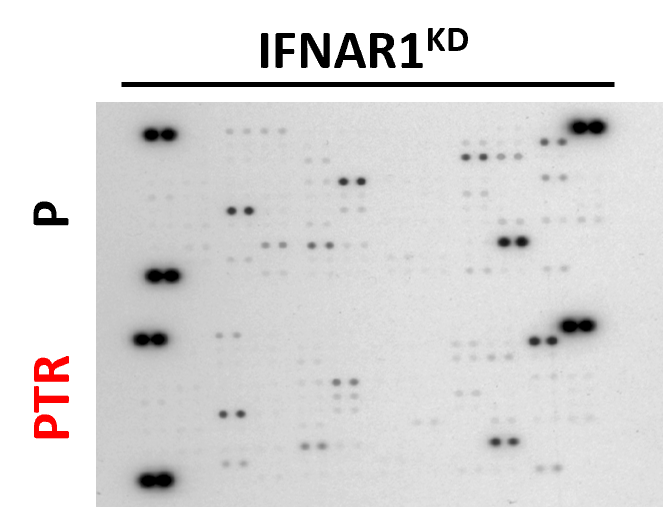

Supplement: Supplementary file 4 — Source data Fig. 2 [file 44319_2024_333_MOESM4_ESM.zip › Figure 2/2C/2C-right no annotations.png]

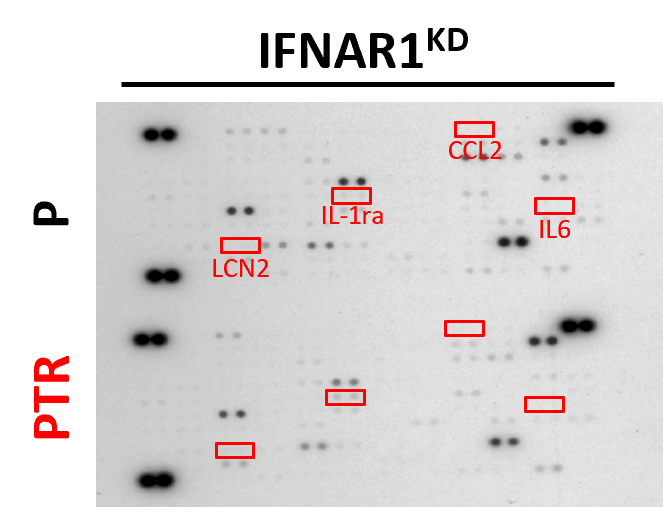

Supplement: Supplementary file 4 — Source data Fig. 2 [file 44319_2024_333_MOESM4_ESM.zip › Figure 2/2C/2C-right.png]

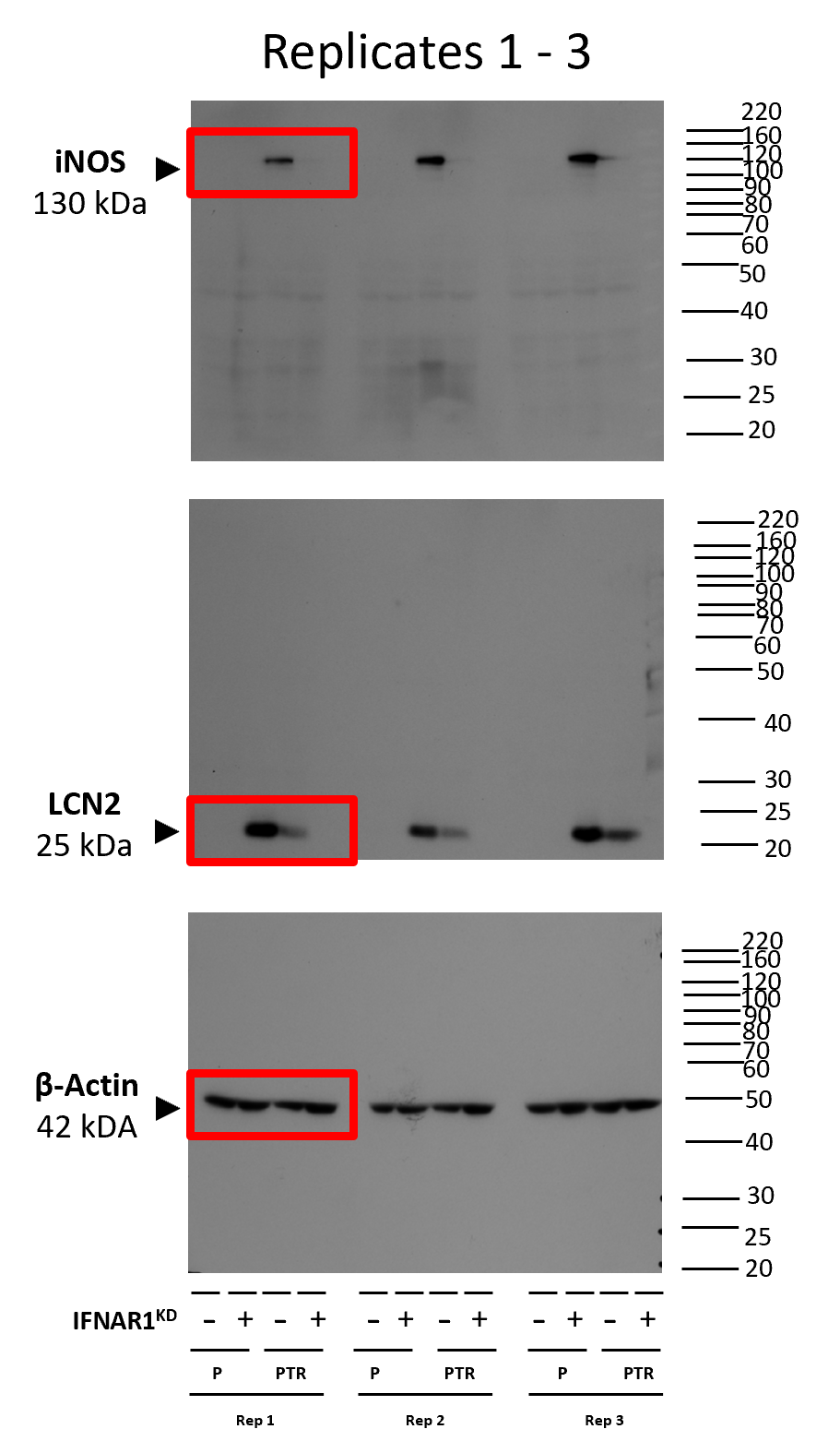

Supplement: Supplementary file 4 — Source data Fig. 2 [file 44319_2024_333_MOESM4_ESM.zip › Figure 2/2E/2E.png]

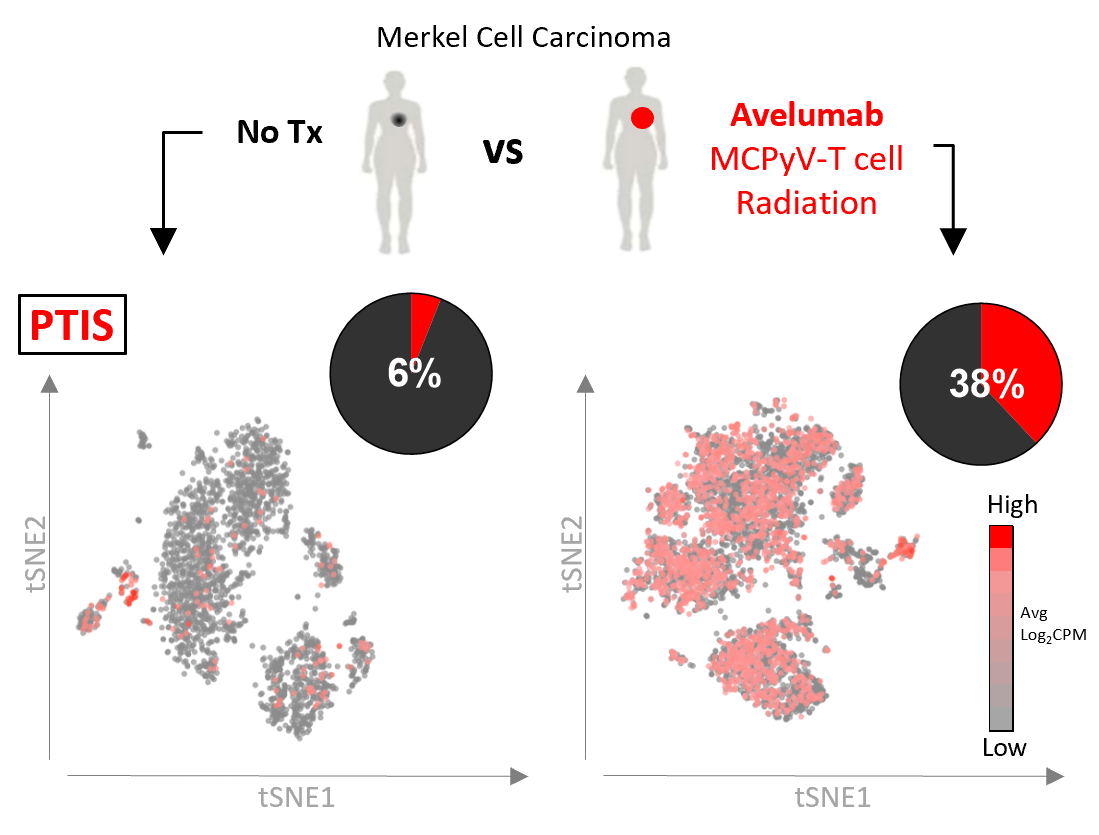

Supplement: Supplementary file 5 — Source data Fig. 3 [file 44319_2024_333_MOESM5_ESM.zip › Figure 3/3E/3E.png]

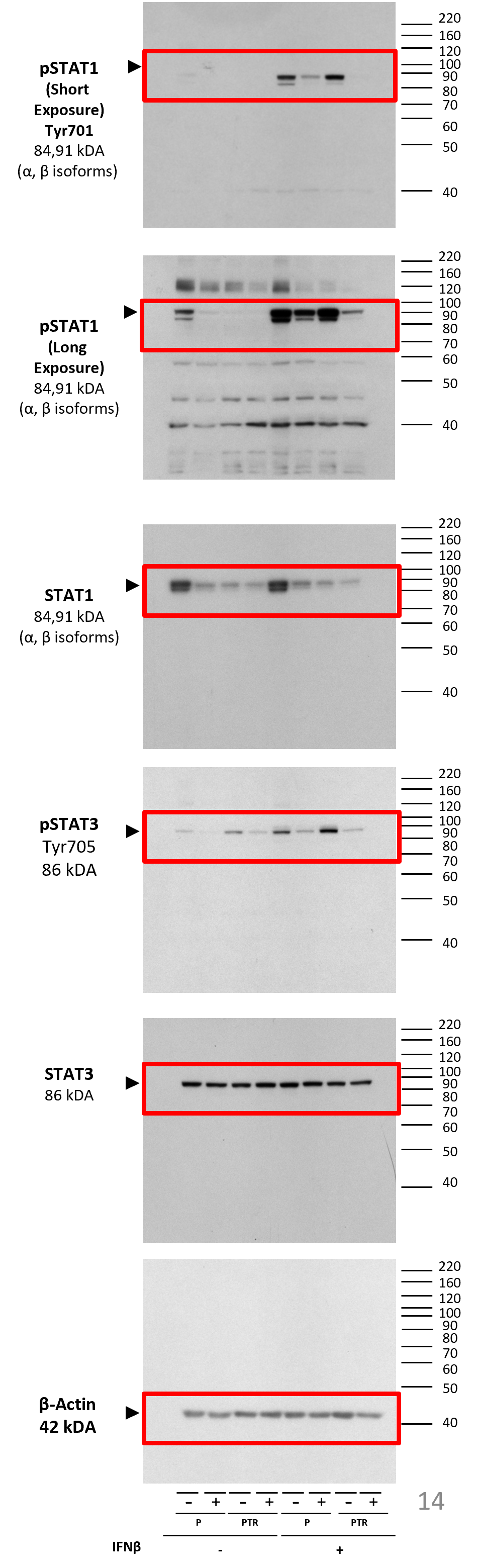

Supplement: Supplementary file 6 — Source data Fig. 4 [file 44319_2024_333_MOESM6_ESM.zip › Figure 4/4F/4F.png]

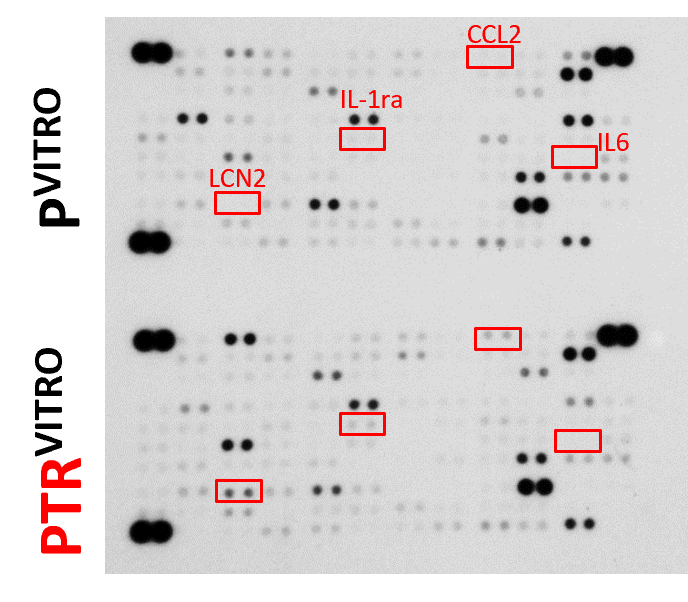

Supplement: Supplementary file 7 — Source data Fig. 5 [file 44319_2024_333_MOESM7_ESM.zip › Figure 5/5A-B/5A-B Annotated.png]

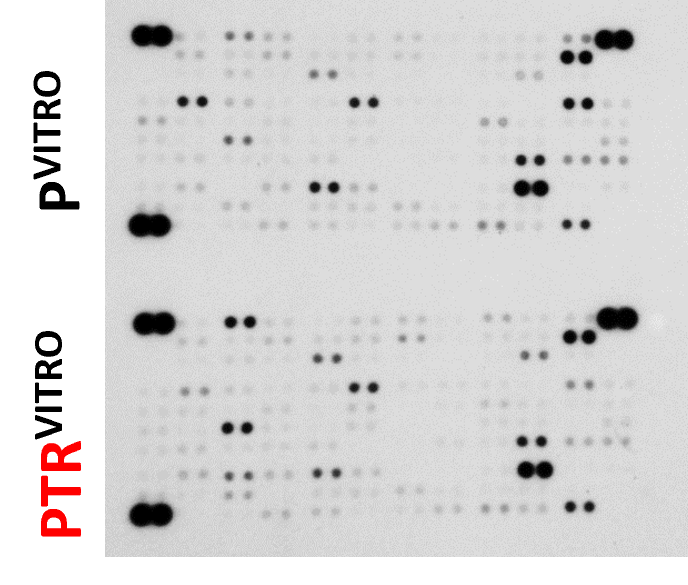

Supplement: Supplementary file 7 — Source data Fig. 5 [file 44319_2024_333_MOESM7_ESM.zip › Figure 5/5A-B/5A-B.png]

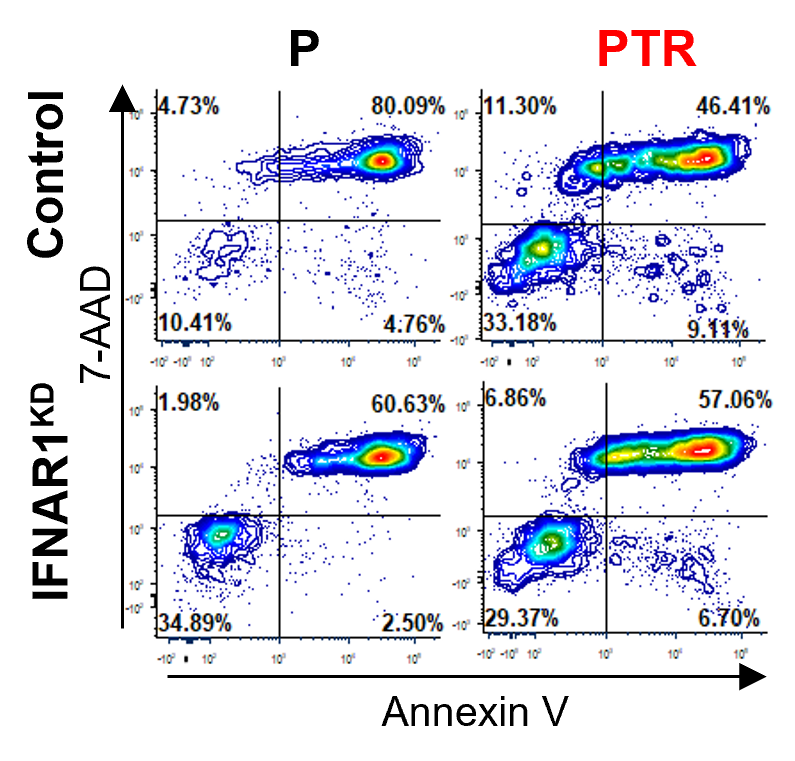

Supplement: Supplementary file 8 — Source data Fig. 6 [file 44319_2024_333_MOESM8_ESM.zip › Figure 6/6C/6C.png]

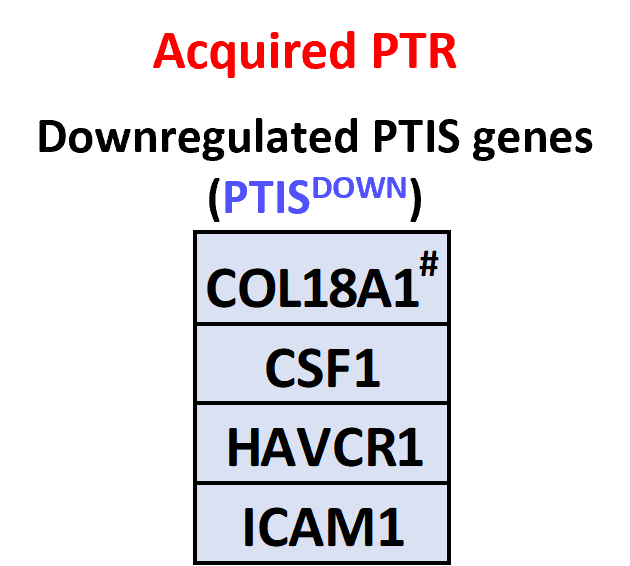

Supplement: Supplementary file 10 — Appendix and EV Figures Source Data [file 44319_2024_333_MOESM10_ESM.zip › Figure EV2/EV2A/EV2A.png]

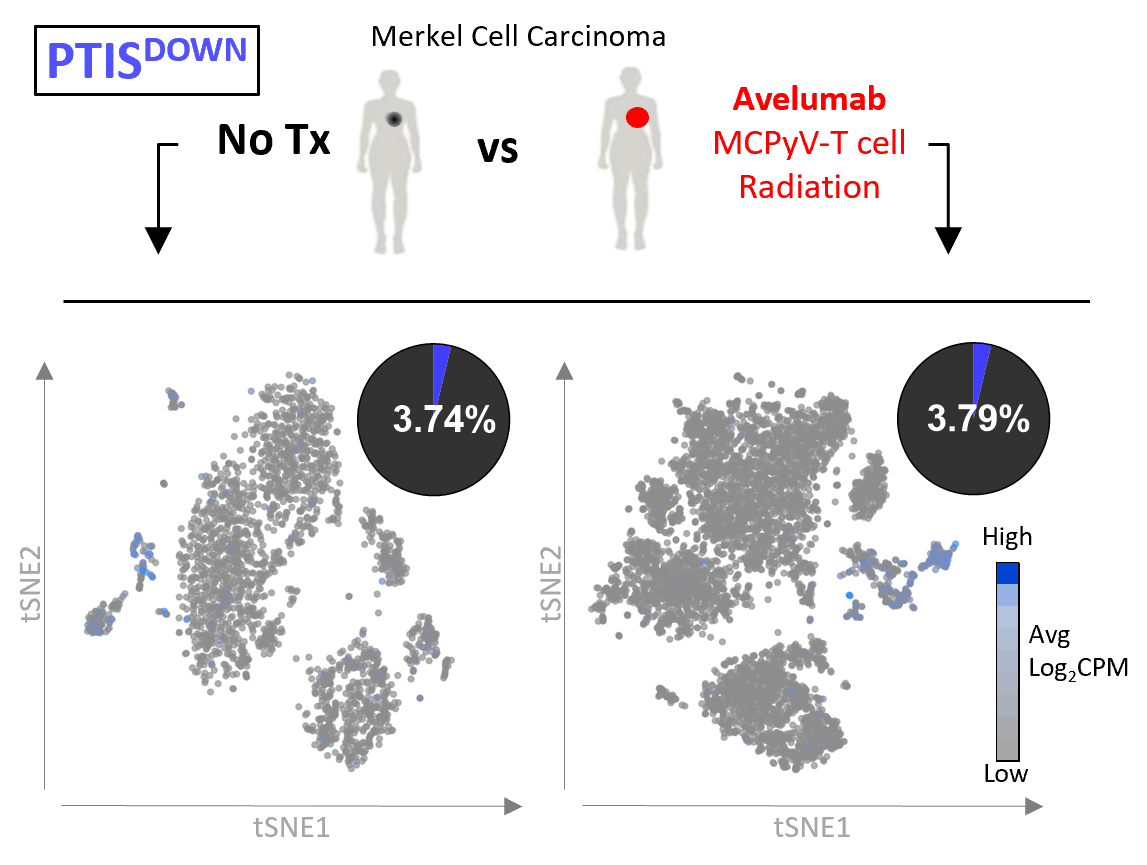

Supplement: Supplementary file 10 — Appendix and EV Figures Source Data [file 44319_2024_333_MOESM10_ESM.zip › Figure EV2/EV2E/EV2E.png]
