# Supplementary material for: Acquired resistance to PD-L1 inhibition enhances a type I IFN-regulated secretory program in tumors
Source: EMBO Rep. 2024 Dec 11;26(2):521–59. doi: 10.1038/s44319-024-00333-0 (PMC11772817; doi:10.1038/s44319-024-00333-0)
Supplement: Supplementary file 11 — Expanded View Figures [file 44319_2024_333_MOESM11_ESM.pdf]

Expanded View Figures

Figure EV1. Identification of an αPD-L1 treatment-induced secretome (PTIS) gene signature.

(**A**) IFNAR1 expression in EMT6-P and -PTR before and after knockdown of IFNAR1 and respective vector controls. Flow cytometry, $n = 3$, statistics performed via two-tailed t-test. Bar graphs show mean ± SD. (**B**) Bar plot summary of cytokine protein array shown as EMT6-PTR compared to parental controls from Fig. 2C. Bars representing mean value of two separate experiments (shown as dots), $n = 2$. (**C**) Heatmap summary of type I IFN regulated proteins in cytokine protein array. (**D**) Table summary of PTIS transcriptomic and proteomic confirmations shown in Fig. 2B–H. Data Information: αPD-L1 Treatment-Induced Secretome (PTIS); αPD-L1 Treatment-Resistant (PTR);Counts per million (CPM); IFNAR1 knockdown ($IFNAR1^{KD}$); Control Knockdown (shCon); Not Tested (NT). $^{*}p \le 0.05$, $^{**}p \le 0.01$, $^{***}p \le 0.001$, $^{****}p \le 0.0001$, for exact p values see Fig. EV1 Source data. All replicates shown represent technical replicates unless otherwise specified. Source data are available online for this figure.

 © The Author(s)

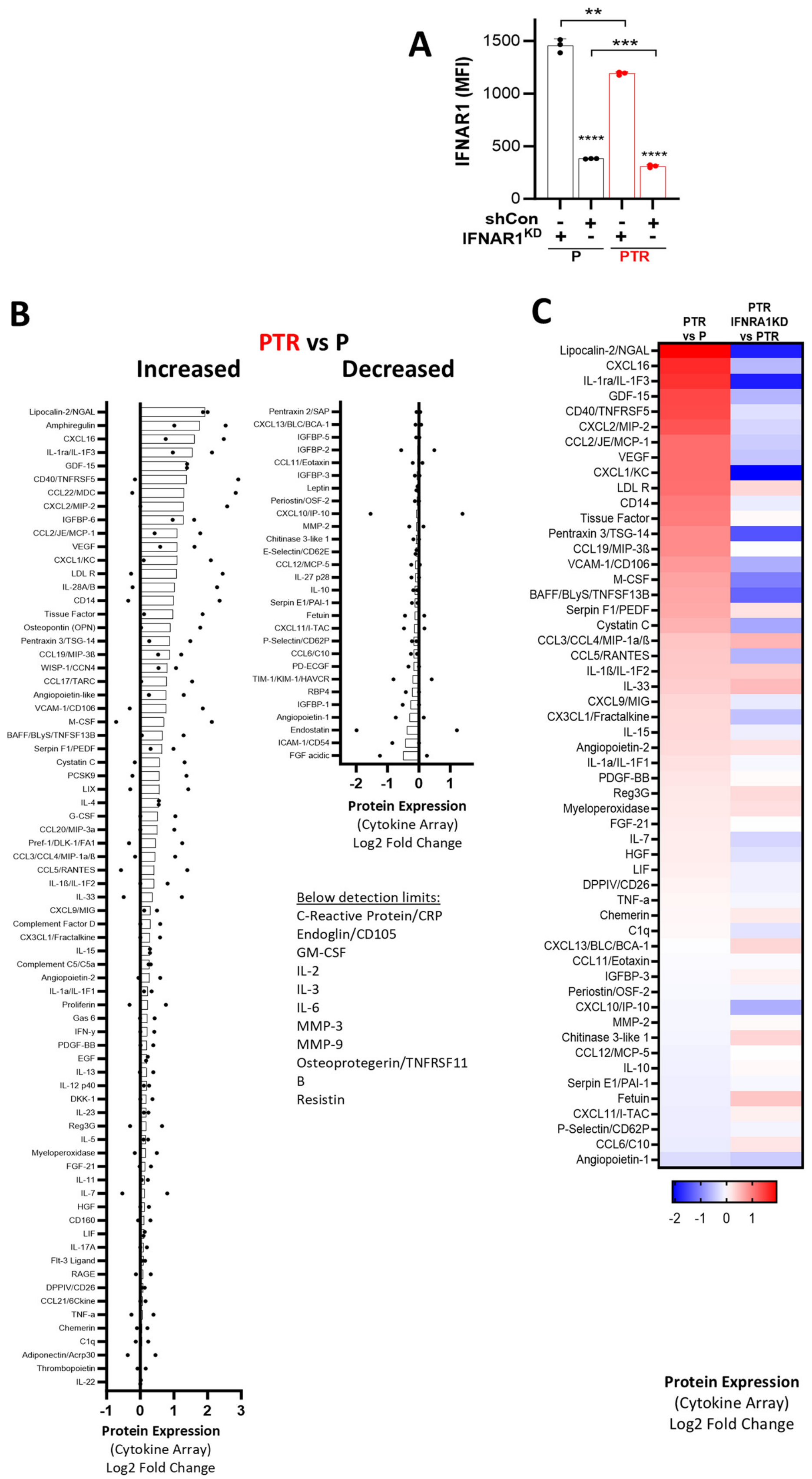

D

PTIS	Confirmation of Increase	
	Transcriptomic	Proteomic
CCL2	●	● (blue)
IL1RN	●	● (blue)
IL6	●	● (red)
LCN2	●	● (blue)
NOS2	●	● (purple)

Methodology
● (black) RNAseq / RT PCR
● (red) ELISA
● (blue) Protein Array
● (purple) Western blot

© The Author(s)

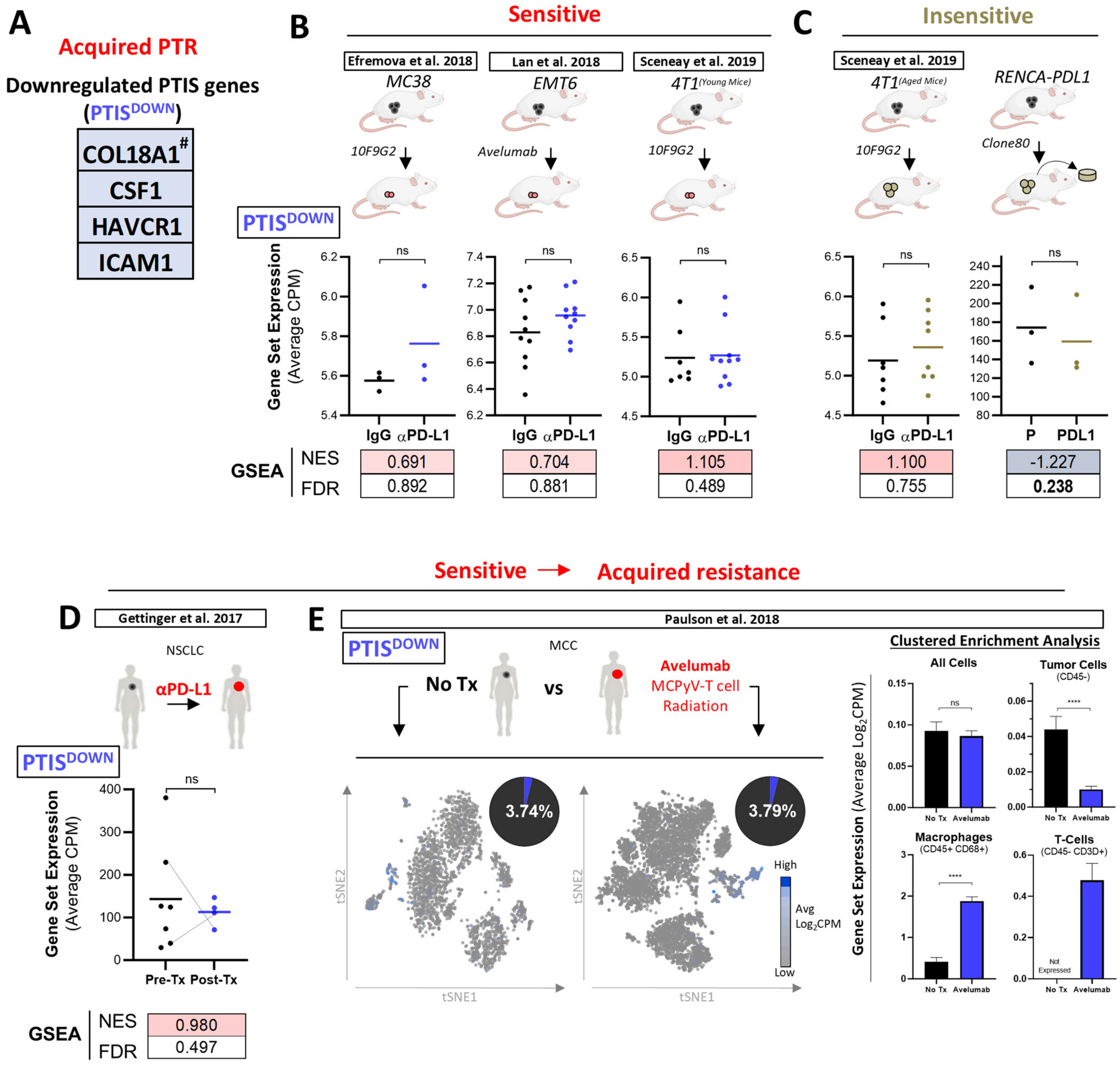
A
Acquired PTR
Downregulated PTIS genes
(PTISDOWN)
COL18A1#
CSF1
HAVCR1
ICAM1
B
Sensitive
Efremova et al. 2018
MC38
10F9G2
Lan et al. 2018
EMT6
Avelumab
Sceneay et al. 2019
4T1(Young Mice)
10F9G2
PTISDOWN
Gene Set Expression (Average CPM)
ns
IgG αPD-L1
GSEA
NES
FDR
0.691
0.892
0.704
0.881
1.105
0.489
C
Insensitive
Sceneay et al. 2019
4T1(Aged Mice)
10F9G2
RENCA-PDL1
Clone80
P PDL1
1.100
0.755
-1.227
0.238
Sensitive → Acquired resistance
D
Gettinger et al. 2017
NSCLC
αPD-L1
PTISDOWN
Gene Set Expression (Average CPM)
Pre-Tx Post-Tx
GSEA
NES 0.980
FDR 0.497
E
Paulson et al. 2018
PTISDOWN
MCC
No Tx
vs
Avelumab
MCPyV-T cell
Radiation
3.74%
3.79%
tSNE1
tSNE2
High
Avg
Log2CPM
Low
Clustered Enrichment Analysis
Gene Set Expression (Average Log2CPM)
All Cells
Tumor Cells (CD45-)
Macrophages (CD45+ CD68+)
T-Cells (CD45- CD3D+)
No Tx Avelumab
Not Expressed

 © The Author(s)

Figure EV2. A downregulated PTIS signature (PTIS^{DOWN}) is variably expressed in clinical and preclinical models sensitive to αPD-L1 treatment.

(**A**) Transcriptome and proteomic analysis were used to generate a signature comprised only of genes significantly *down*regulated (PTIS^{DOWN}) in EMT6-PTR cells. The 4-gene PTIS^{DOWN} was comprised primarily of IFN-regulated genes (3 out of 4 total). (**B**, **C**) PTIS^{DOWN} was measured in published preclinical datasets described in Fig. 3B. In αPD-L1 treatment-*sensitive* and -*insensitive* tumor models, enrichment for the PTIS^{DOWN} was variable, with no significant signature expression trends found (as measured by CPM). Only 1 of 5 models (*insensitive;* RENCA-PDL1) showed significant negative GSEA enrichment of the PTIS^{DOWN} signature. *P* values representative of statistics performed via two-tailed t-test. (**B**) Analysis of PTIS^{DOWN} expression in αPD-L1 treatment-*sensitive* tumor models used data from (Lan et al, 2018; Sceneay et al, 2019; Efremova et al, 2018) (Data ref: Lan et al, 2018; Data ref: Sceneay et al, 2019; Data ref: Efremova et al, 2018). Data is compared to vehicle/IgG-treated controls. Central line depicts the median, $n = 3$–10 biological replicates. (**C**) Analysis of PTIS^{DOWN} expression in αPD-L1 treatment-*insensitive* tumor models used data from (Sceneay et al, 2019) (Data ref: Sceneay et al, 2019) and RENCA-PDL1 model from this study. Central line depicts the median, $n = 3$–8 biological replicates. (**D**, **E**) Using published bulk and single cell RNAseq clinical datasets taken from tumor biopsies of αPD-L1 treatment-*sensitive* patients, PTIS^{DOWN} expression was assessed by average CPM expression and GSEA comparisons. *P* values representative of statistics performed via two-tailed t-test. (**D**) Analysis of PTIS^{DOWN} signature showed decreases in NSCLC after treatment significance not reached). Bulk RNAseq from (Gettinger et al, 2017) (Data ref: Gettinger et al, 2017) to compare Pre- and Post-treatment (Tx) tumor sample comparisons (Gray lines indicate matched Pre- and Post-tx samples). Central line depicts the median, $n = 4$–7 biological replicates. (**E**) Clustered analysis of MCC avelumab-treated samples showed variable, but significant PTIS^{DOWN} changes in enriched tumor cell (decreased expression) and macrophage clusters (increased expression). Single-cell RNAseq data obtained from (Paulson et al, 2018) (Data ref: Paulson et al, 2018) represent untreated (No-Tx) or treated (avelumab, MCPyV-T cell, radiation) tumor samples ($n = 1$) with tSNE plots (left) representing average log_2CPM expression of PTIS in whole dataset, and bar graphs (right) representing clustered enrichment analysis populations identified by markers for tumors (CD45−), macrophages (CD68+), and T cells (CD3D+). Tumor sample that received No-Tx was compared to treated. Data Information: αPD-L1 Treatment-Induced Secretome involving only downregulated genes (PTIS^{DOWN}); αPD-L1 Treatment-Resistant (PTR); Counts per million (CPM); Gene set enrichment analysis (GSEA); False Discovery Rate (FDR); Gene Expression Omnibus (GEO); GEO Series records (GSE); database of Genotypes and Phenotypes (dbGaP); t-distributed stochastic neighbor embedding (tSNE); Treatment (Tx); non-small cell lung carcinoma (NSCLC); Merkel cell carcinoma (MCC). Bar graphs show mean ± SEM. Scatter dot plot central line show mean. Bolded numbers for GSEA represent FDR < 0.25 (see Methods). *$p \le 0.05$, **$p \le 0.01$, ***$p \le 0.001$, ****$p \le 0.0001$, except for GSEA as indicated above, for exact *p* values see Fig. EV2 Source data. Source data are available online for this figure.

© The Author(s)

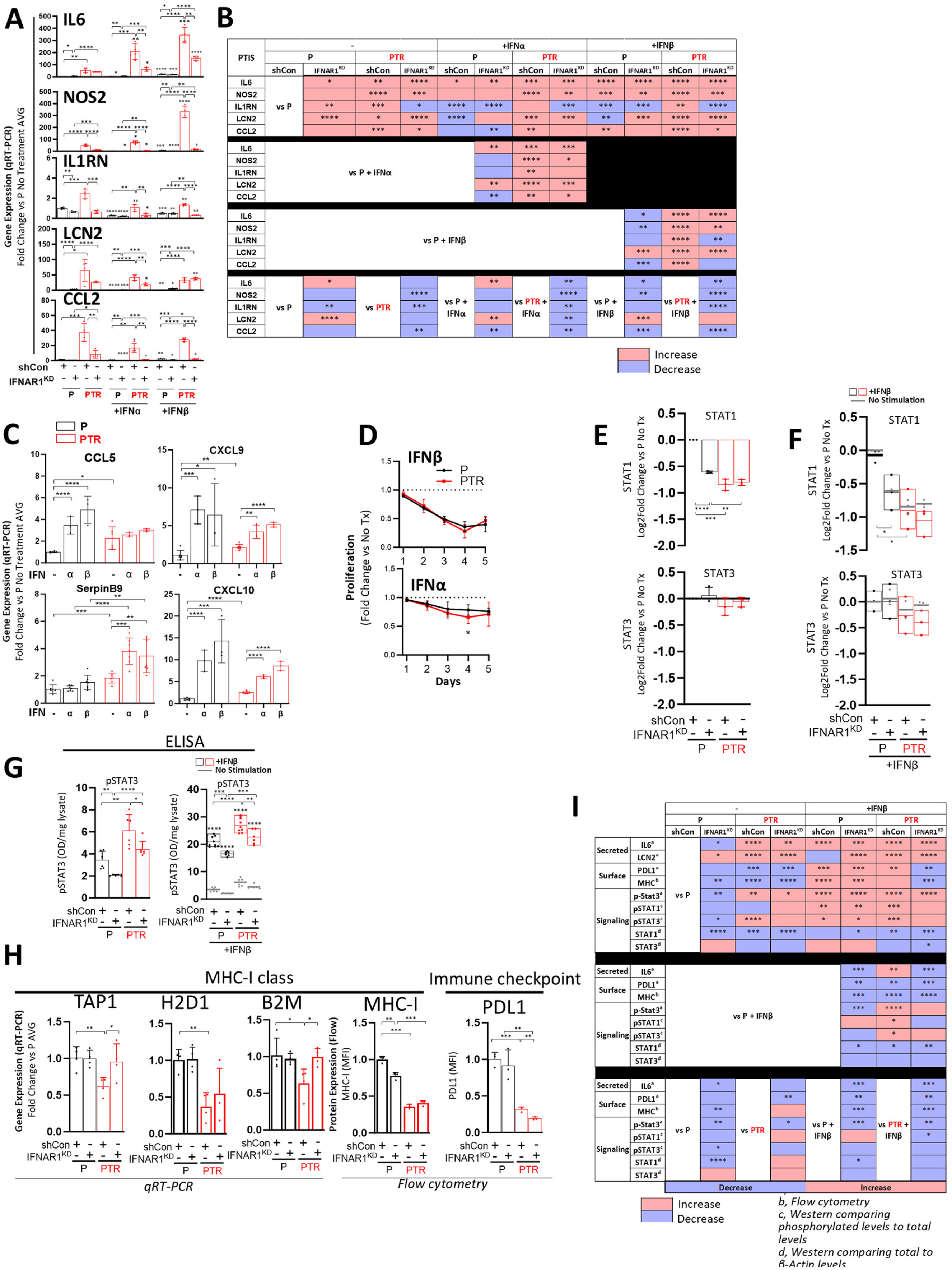
A
IL6
NOS2
IL1RN
LCN2
CCL2
Gene Expression (qRT-PCR)
Fold Change vs P No Treatment AVG
shCon
IFNAR1KD
P PTR P PTR P PTR
+IFNα +IFNβ
B
PTIS
- +IFNα +IFNβ
P PTR
shCon IFNAR1KD
vs P
vs P + IFNα
vs P + IFNβ
vs PTR
vs P + IFNα
vs PTR + IFNα
vs P + IFNβ
vs PTR + IFNβ
Increase
Decrease
C
P
PTR
CCL5
CXCL9
SerpinB9
CXCL10
IFN - α β - α β
Gene Expression (qRT-PCR)
Fold Change vs P No Treatment AVG
D
IFNβ
IFNα
P
PTR
Proliferation
(Fold Change vs No Tx)
Days
E
STAT1
STAT3
Log2Fold Change vs P No Tx
shCon
IFNAR1KD
P PTR
F
+IFNβ
No Stimulation
STAT1
STAT3
+IFNβ
G
ELISA
pSTAT3
pSTAT3 (OD/mg lysate)
+IFNβ
No Stimulation
H
MHC-I class
Immune checkpoint
TAP1
H2D1
B2M
MHC-I
PDL1
Gene Expression (qRT-PCR)
Fold Change vs P AVG
Protein Expression (Flow)
MHC-I (MFI)
PDL1 (MFI)
qRT-PCR
Flow cytometry
I
- +IFNβ
Secreted
Surface
Signaling
IL6a
LCN2a
PDL1a
MHCb
p-Stat3a
pSTAT1c
pSTAT3c
STAT1d
STAT3d
vs P
vs P + IFNβ
vs PTR
vs P + IFNβ
vs PTR + IFNβ
Decrease
Increase
b, Flow cytometry
c, Western comparing phosphorylated levels to total levels
d, Western comparing total to β-Actin levels

 © The Author(s)

Figure EV3. IFNs modulate PTIS, STAT signaling, and surface ISGs in PTR cells.

(**A**) PTIS factor expression in EMT6-P and -PTR cells before and after knockdown of IFNAR1^{KD}, and after IFN α/β stimulation shown as bar plots. Full datasets for Fig. 4B, C, D. qRT-PCR, statistics performed via two-tailed t-test, $n = 4$. (**B**) Table summarizing two-tailed t-test statistical comparisons for qRT-PCR data shown in Fig. 4B, C, D. (**C**) ISG expression in EMT6-P and -PTR cells after IFN α/β stimulation shown as bar plots. qRT-PCR, statistics performed via two-tailed t-test, $n = 3$–7 (1–2 biological replicates). (**D**) EMT6-P and PTR were treated with IFNα (left), and IFNβ (right) for 5 days and proliferation measured daily by MTS, statistics performed via two-tailed t-test, $n = 10$. (**E**, **F**) Densitometry quantification of western blots shown in (Fig. 4F) representing relative (top) total STAT1 and (bottom) total STAT3 levels compared to total β-Actin levels (**E**) at baseline and (**F**) after IFNβ stimulation. Statistics performed via two-tailed t-test. Boxes indicate range between minimum and maximum, central line depicts the mean, $n = 3$. (**G**) pSTAT3 expression in lysates of EMT6-P and -PTR before and after knockdown of IFNAR1^{KD}, and after IFNβ stimulation. ELISA, statistics performed via two-tailed t-test. Boxes indicate range between minimum and maximum, central line depicts the mean, $n = 6$–9. (**H**) Antigen presentation machinery expression in EMT6-P and -PTR cells before and after knockdown of IFNAR1^{KD} shown as bar plots. Full dataset for Fig. 4I, J. qRT-PCR and flow cytometry as indicated, statistics performed via two-tailed t-test, $n = 3$–4. (**I**) Table summarizing two-tailed t-test statistical comparisons for data shown in Figs. 4 and EV3. Superscripts: a—ELISA, b—Flow cytometry, c—western comparing phosphorylated levels to total levels, d—western comparing total to β-Actin levels. Data Information: Parental (P); αPD-L1 Treatment-Resistant (PTR); Conditioned Media (CM); Mean Fluorescent Intensity (MFI); IFN stimulated genes (ISGs); IFNAR1 knockdown (IFNAR1^{KD}); shRNA vector control (shCon); Cells were treated with 10 ng/ml of IFNs and collected after 15 min for STAT1/3 westerns and ELISA; for proliferation experiments cells were treated with 10 ng/ml of IFNs starting on day 0 and treatment and fresh media was replaced on day 3. Bar graphs and line graphs show mean ± SD. Box plots indicate range between minimum and maximum, central line depicts the mean. $^*p \le 0.05$, $^{**}p \le 0.01$, $^{***}p \le 0.001$, $^{****}p \le 0.0001$ indicate significance compared untreated controls unless otherwise shown (lines), for exact p values see Fig. EV3 Source data. All replicates shown represent technical replicates unless otherwise specified. Source data are available online for this figure.

© The Author(s)

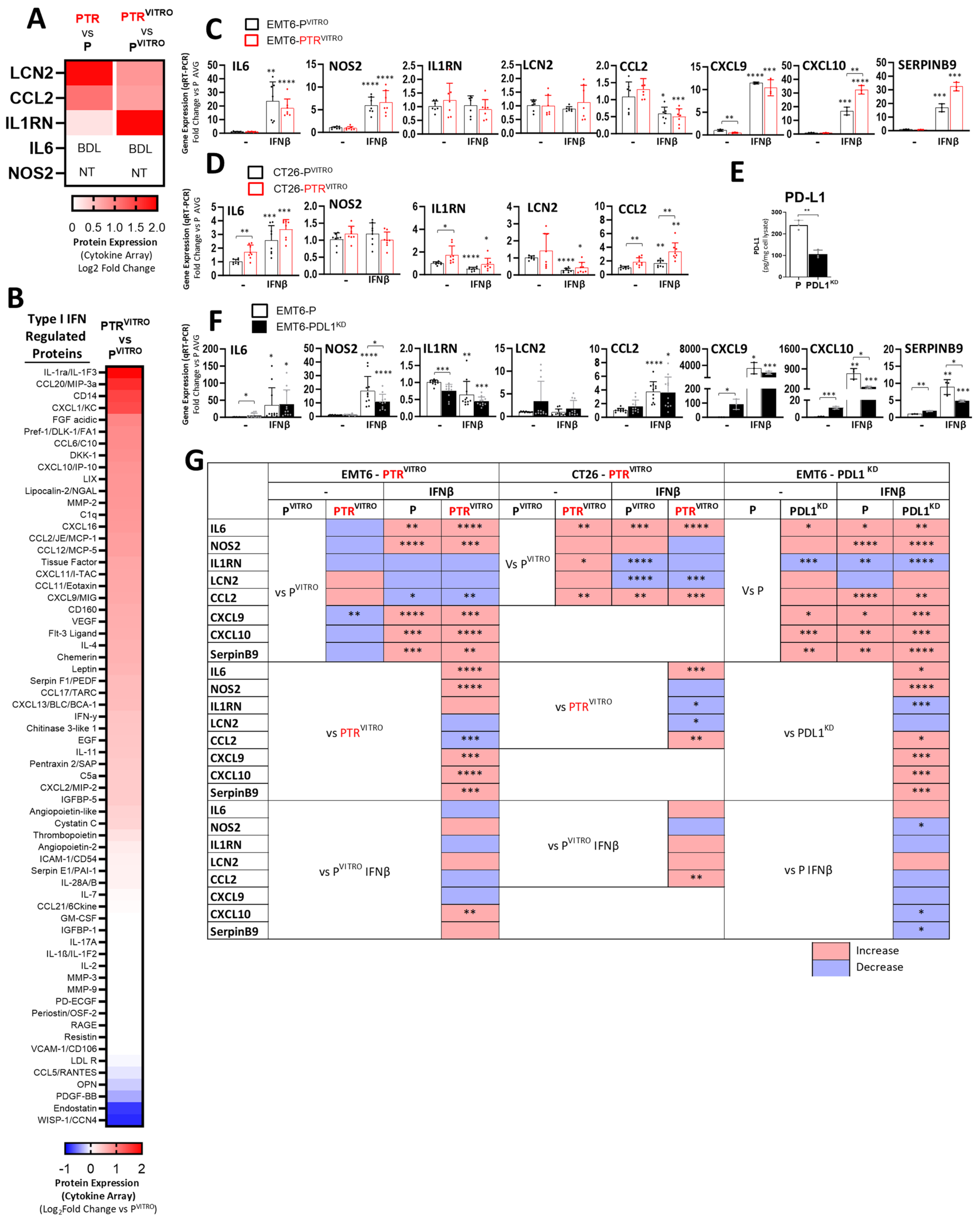
A
PTR vs P
PTRVITRO vs PVITRO
LCN2
CCL2
IL1RN
IL6
NOS2
BDL
NT
Protein Expression
(Cytokine Array)
Log2 Fold Change
B
Type I IFN Regulated Proteins
PTRVITRO vs PVITRO
Protein Expression
(Cytokine Array)
(Log2Fold Change vs PVITRO)
C
EMT6-PVITRO
EMT6-PTRVITRO
Gene Expression (qRT-PCR)
Fold Change vs P AVG
IL6
NOS2
IL1RN
LCN2
CCL2
CXCL9
CXCL10
SERPINB9
IFNβ
D
CT26-PVITRO
CT26-PTRVITRO
E
PD-L1
PD-L1 (pg/mg cell lysate)
P
PDL1KD
F
EMT6-P
EMT6-PDL1KD
G
EMT6 - PTRVITRO
CT26 - PTRVITRO
EMT6 - PDL1KD
vs PVITRO
vs PTRVITRO
vs PVITRO IFNβ
Vs P
vs PDL1KD
vs P IFNβ
Increase
Decrease

© The Author(s)

Figure EV4. PTIS expression in PTR^{VITRO} and $PD\text{-}L1^{KD}$ models.

(**A**) Heatmap summary of PTIS factors in cytokine protein array for EMT6-PTR cells relative to -P controls (Fig. 2A) and EMT6-PTR^{VITRO} cells relative to -P^{VITRO} controls (Fig. 5A). (**B**) Heatmap summary of type I IFN regulated protein changes in cytokine protein array for EMT6-PTR cells relative to -P controls (Fig. 5A) and EMT6-PTR^{VITRO} cells relative to -P^{VITRO} controls (Fig. 2A). (**C**) PTIS and various ISG expression in EMT6-P^{VITRO} and -PTR^{VITRO} cells treated with IFNβ shown as relative to P controls and represented as bar graphs. Full dataset for Fig. 5F. qRT-PCR, statistics performed via two-tailed t-test, $n = 3$–7 (1–2 biological replicates). (**D**) PTIS expression in CT26-P^{VITRO} and -PTR^{VITRO} cells treated with IFNβ shown as relative to P controls and represented as bar graphs. Full dataset for Fig. 5G. qRT-PCR, statistics performed via two-tailed t-test, $n = 8$ (2 biological replicates). (**E**) PD-L1 expression in cell lysates of EMT6-P and EMT6–PD-$L1^{KD}$ cells. ELISA, statistics performed via two-tailed t-test, $n = 3$. (**F**) PTIS and various ISG expression in EMT6-shCon and -$PDL1^{KD}$ cells treated with IFNβ shown as relative to P controls and represented as bar graphs. Full dataset for Fig. 5H. qRT-PCR, statistics performed via two-tailed t-test, $n = 3$–11 (1–3 biological replicates). (**G**) Tables summarizing two-tailed t-test statistical comparisons for qRT-PCR data shown in Fig. 5E, F, G, EV4C, EV4D, and EV4F. Data Information: Parental (P); αPD-L1 Treatment-Resistant (PTR); PD-L1 knockdown ($PDL1^{KD}$) Bar graphs show mean ± SD. *$p \leq 0.05$, **$p \leq 0.01$, ***$p \leq 0.001$, ****$p \leq 0.0001$ compared to vector controls unless noted otherwise, for exact p values see Fig. EV4 Source data. All replicates shown represent technical replicates unless otherwise specified. Source data are available online for this figure.

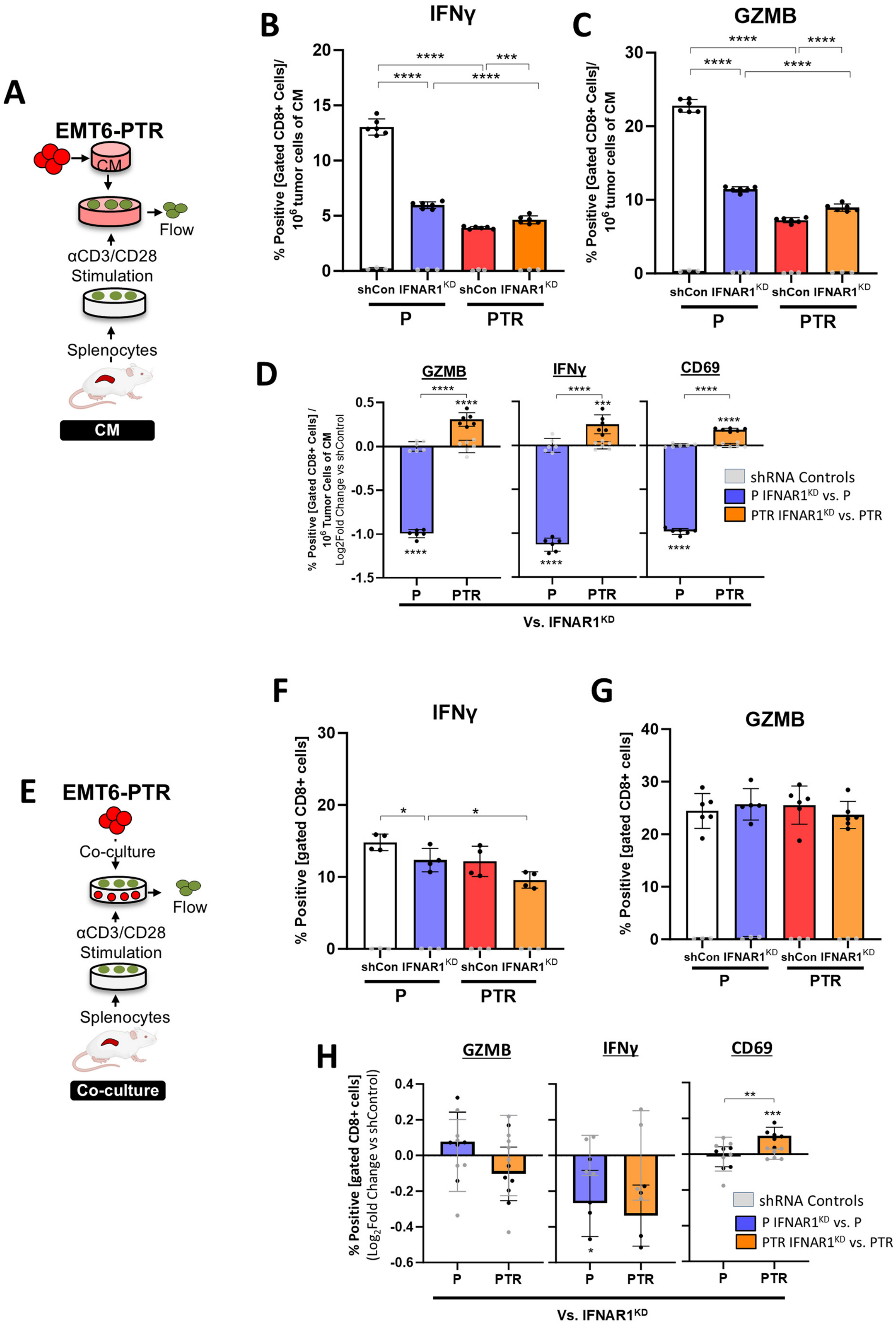
A
EMT6-PTR
CM
Flow
αCD3/CD28
Stimulation
Splenocytes
CM
B
IFNγ
% Positive [Gated CD8+ Cells]/
10^6 tumor cells of CM
shCon IFNAR1KD shCon IFNAR1KD
P
PTR
C
GZMB
% Positive [Gated CD8+ Cells]/
10^6 tumor cells of CM
shCon IFNAR1KD shCon IFNAR1KD
P
PTR
D
GZMB
IFNγ
CD69
% Positive [Gated CD8+ Cells] /
10^6 Tumor Cells of CM
Log2Fold Change vs shControl
shRNA Controls
P IFNAR1KD vs. P
PTR IFNAR1KD vs. PTR
P PTR P PTR P PTR
Vs. IFNAR1KD
E
EMT6-PTR
Co-culture
Flow
αCD3/CD28
Stimulation
Splenocytes
Co-culture
F
IFNγ
% Positive [gated CD8+ cells]
shCon IFNAR1KD shCon IFNAR1KD
P
PTR
G
GZMB
% Positive [gated CD8+ cells]
shCon IFNAR1KD shCon IFNAR1KD
P
PTR
H
GZMB
IFNγ
CD69
% Positive [gated CD8+ cells]
(Log2Fold Change vs shControl)
shRNA Controls
P IFNAR1KD vs. P
PTR IFNAR1KD vs. PTR
P PTR P PTR P PTR
Vs. IFNAR1KD

 © The Author(s)

Figure EV5. Splenocyte activation following incubation with PTR cells and conditioned media.

(**A**) Schematic of Balb/c-derived splenocyte proliferation and activation following incubation EMT6-P and -PTR CM for experiments in (**B–E**). (**B**, **C**) CD8+ splenocyte activation marker (**B**) IFNγ and (**C**) Granzyme B after co-incubation with CM derived from EMT6-P and -PTR control and respective IFNAR1^{KD} variants. Flow cytometry, statistics performed via two-tailed t-test, $n = 3–6$. (**D**) Log_2 fold change analysis of CD8+ splenocyte activation markers (Granzyme B, IFNγ, CD69) after co-incubation with EMT6-P and -PTR-IFNAR1^{KD} variant CM compared to respective controls. Flow cytometry, statistics performed via two-tailed t-test, $n = 3–6$. (**E**) Schematic of Balb/c-derived splenocyte proliferation and activation following incubation EMT6-P and -PTR CM for experiments in (**F–H**). (**F**, **G**) CD8+ splenocyte activation marker (**F**) IFNγ (**G**) Granzyme B after co-culture with EMT6-P and -PTR control and respective IFNAR1^{KD} variants. Flow cytometry, statistics performed via two-tailed t-test, $n = 3–6$. (**H**) Log_2 fold change analysis of CD8+ splenocyte activation markers (Granzyme B, IFNγ, CD69) after co-culture with EMT6-P and -PTR-IFNAR1^{KD} variant compared to respective controls, statistics performed via two-tailed t-test, $n = 4–6$. Data Information: Parental (P); αPD-L1 Treatment-Resistant (PTR); IFNAR1 knockdown (IFNAR1^{KD}); Conditioned Media (CM); Granzyme B (GZMB). Bar graphs show mean ± SD. *$p \leq 0.05$, **$p \leq 0.01$, ***$p \leq 0.001$, ****$p \leq 0.0001$ compared to vector controls unless noted otherwise, for exact *p* values see Fig. EV5 Source data. All replicates shown represent technical replicates unless otherwise specified. Source data are available online for this figure.

© The Author(s)
